# Account of Diseases in an Eastern District of London, from May 20, to June 20, 1803

**Published:** 1803-07-01

**Authors:** 


*Account of Diseases in an Eastern District of London, from May 20, to June 20, 1803.*

| | |
|---|---|
| ACUTE DISEASES. | |
| Typhus Mitior - - - - | 6 |
| Scarlatina Anginosa - - | 4 |
| Ophthalmia - - - - - | 2 |
| Acute Rheumatism - - | 6 |
| CHRONIC DISEASES. | |
| Cough - - - - - - - - | 7 |
| Dyspnœa - - - - - - | 4 |
| Cough & Dyspnœa - - | 9 |
| Phthisis Pulmonalis - - | 4 |
| Hydrothorax - - - - - | 3 |
| Ascites - - - - - - - | 2 |
| Anasarca - - - - - - - | 4 |
| Cephalalgia - - - - - | 7 |
| Gastrodynia - - - - - | 10 |
| Dyspepsia - - - - - - | 6 |
| Enterodynia - - - - - | 3 |
| Procidentia Ani - - - | 2 |
| Hæmorrhois - - - - - | 4 |
| Diarrhœa - - - - - - | 3 |
| Obstipatio - - - - - - | 5 |
| Dysuria - - - - - - - | 2 |
| Hypochondriasis - - - - | 4 |
| Hysteria - - - - - - | 3 |
| Fluor Albus - - - - - | 7 |
| Rheumatismus Chronicus | 10 |
| PUERPERAL DISEASES. | |
| Menorrhagia Lochialis - | 4 |
| Enuresis - - - - - - - | 1 |
| Peritonitis - - - - - - | 3 |
| Mastodynia - - - - - | 6 |
| INFANTILE DISEASES. | |
| Convulsio - - - - - - | 2 |
| Vermes - - - - - - - | 4 |
| Ophthalmia - - - - - | 3 |
| Aphthæ - - - - - - | 4 |
| Herpes - - - - - - - | 7 |

The late epidemic seems now to have entirely subsided, and the general state of health to have much improved. There have, however, been a few instances of typhus, but it has appeared under a mild form, and has generally terminated favourably.

Scarlatina anginosa has prevailed amongst children, though the symptoms have not been very severe. The tonsils were slightly swelled and inflamed, and deglutition was attended with some difficulty. In a little time an efflorescence appeared on the skin, and general symptoms of fever prevailed. The disease appearing in this mild form, very soon gave way to the usual treatment.

The disease, in every instance at present referred to, was followed by general anasarcous swellings; these, however, disappeared in a few days, and left the patients in perfect health.

